# Drug Metabolism and Pharmacokinetics of Oxazolo[4,5-*c*]quinoline Analogs as Novel Interleukin-33 Inhibitors

**DOI:** 10.3390/pharmaceutics17091153

**Published:** 2025-09-03

**Authors:** Hayoung Jeon, Geonhee Jang, Min-A Ban, Sang-Hyun Son, Youngjoo Byun, Kiho Lee

**Affiliations:** 1 College of Pharmacy, Korea University, Sejong 30019, Republic of Korea; 2020020911@korea.ac.kr (H.J.); geonhee416@naver.com (G.J.); ba0915@naver.com (M.-A.B.); 2 Azcuris Co., Ltd., 2511 Sejong-ro, Sejong 30019, Republic of Korea; sonsh@azcuris.com; 3 Interdisciplinary Major Program in Innovative Pharmaceutical Sciences, Korea University, Sejong 30019, Republic of Korea

**Keywords:** Interleukin-33, IL-33 inhibitor, oxazolo[4,5-*c*]quinoline, pharmacokinetics, drug metabolism, metabolic stability, metabolite identification, KB-1517, KB-1518

## Abstract

**Background/Objectives**: Interleukin-33 (IL-33) is crucial in immune-mediated diseases like asthma. Targeting the IL-33/ST2 pathway holds therapeutic promise. This study characterized the pharmacokinetics (PK) and metabolism of KB-1517 and KB-1518, new oxazolo[4,5-*c*]quinoline IL-33 inhibitors. **Methods**: PK studies were conducted in male ICR mice following intravenous (IV) and oral (PO) administration. In vitro metabolic stability and metabolite identification were assessed using human and mouse liver S9 fractions supplemented with cofactors (NADPH, UDPGA, PAPS, GSH). Plasma and incubation samples were analyzed using validated LC-MS/MS methods. **Results**: KB-1517 exhibited slow absorption/elimination and high apparent oral bioavailability (>100%) post-PO, with an unusually late increase in plasma concentration after IV dosing, hindering terminal parameter calculation. KB-1518 showed low clearance post-IV but suffered from low oral bioavailability (~14%). Both compounds demonstrated high in vitro metabolic stability (t_½_ > 60 min) in both human and mouse liver S9 fractions. Primary metabolism involved phase I oxidation (N-oxidation and N-demethylation), yielding several metabolites identified in vitro and confirmed in vivo. Some species differences in metabolite profiles were observed. **Conclusions**: KB-1517 and KB-1518 are promising, metabolically stable IL-33 inhibitor lead compounds with distinct PK profiles. KB-1517’s complex kinetics suggest potential sustained exposure but require further elucidation. KB-1518’s low oral bioavailability necessitates further optimization. These ADME findings provide a critical foundation for their continued optimization and development.

## 1. Introduction

The incidence of immune-mediated diseases, such as asthma and atopic dermatitis, has been increasing due to rapid societal changes and environmental factors [1,2]. Asthma, a chronic childhood condition characterized by symptoms like shortness of breath and coughing, affects over 300 million people worldwide [3,4]. It is induced by allergens, viral infections, oxidative stress, and air pollution. Atopic dermatitis, another common allergic condition, is triggered by a combination of genetic and environmental factors and is associated with high IgE levels in over 80% of patients [5,6,7]. Both conditions are chronic and can lead to other allergic diseases, including asthma and allergic rhinitis.

The pathogenesis of these diseases involves a T helper 2 (Th2) cell-mediated cytokine response [8,9]. Th2 cells initiate allergic immune reactions, producing cytokines such as IL-4, IL-5, and IL-13, which mediate IgE class switching, eosinophil infiltration, and airway hyperresponsiveness (AHR) [10,11,12]. IL-33, a cytokine derived from epithelial cells, plays a critical role in amplifying Th2 responses by binding to its receptor ST2 on immune cells [13,14,15,16]. This IL-33/ST2 signaling pathway is key in promoting allergic inflammation, including the activation of mast cells, eosinophils, and other immune cells, contributing to the inflammatory cascade in diseases like asthma and atopic dermatitis [17,18,19].

**Figure 1:**
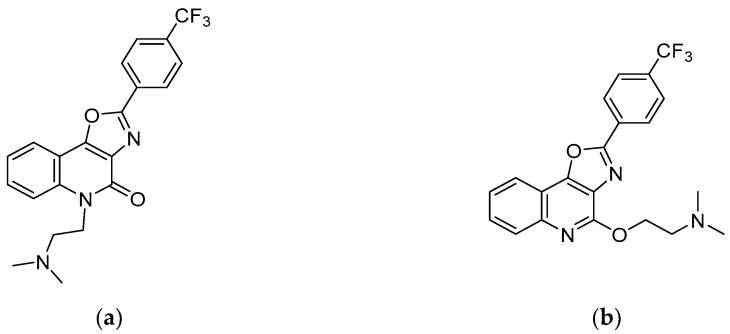
Chemical structures of KB-1517 (**a**) and KB-1518 (**b**).

Given the limitations of current treatments, such as steroids, which have long-term side effects and cannot address the root cause of the diseases [20,21], there is a growing demand for alternative therapies [22,23]. The development of small-molecule inhibitors targeting the IL-33/ST2 interaction holds promise as a potential treatment for these allergic diseases [24,25]. This research focuses on characterizing the pharmacokinetic and metabolic properties of KB-1517 and KB-1518 (Figure 1), two new oxazolo[4,5-*c*]quinoline derivatives recently identified as first-in-class IL-33 inhibitors [26]. These compounds effectively block the IL-33/ST2 interaction, suppress IL-6 production in mast cells in vitro [27,28], and demonstrate efficacy in preclinical models of allergic inflammation [29,30]. Here, we investigate the absorption, distribution, metabolism, and excretion (ADME) properties of KB-1517 and KB-1518, including their pharmacokinetic behavior in mice, in vitro metabolic stability, and major metabolic pathways, to provide insights into their therapeutic potential for treating allergic diseases.

## 2. Materials and Methods

### 2.1. Materials

5-(2-(dimethylamino)ethyl)-2-(4-(trifluoromethyl)phenyl)oxazolo[4,5-*c*]quinolin-4(5H)-one (KB-1517; MW: 401.39) and N,N-dimethyl-2-((2-(4-(trifluoromethyl)phenyl)oxazolo[4,5-*c*]quinolin-4-yl)oxy)ethan-1-amine (KB-1518; MW: 401.39) were synthesized as described previously [26]. Dimethyl sulfoxide (DMSO), β-nicotinamide adenine dinucleotide phosphate (NADPH), diclofenac (internal standard), uridine 5’-diphosphoglucuronic acid (UDPGA), 3’-phosphoadenosine-5’-phosphosulfate (PAPS), reduced glutathione (GSH), tris-HCl, MgCl_2_, alamethicin, propranolol, ranitidine, antipyrine, and itraconazole were purchased from Sigma-Aldrich Korea, Co., Ltd. (Yongin, Gyeonggi-do, Republic of Korea). Benzydamine hydrochloride was purchased from Tokyo Chemical Industry Co., Ltd. (Yangcheon-gu, Seoul, Republic of Korea). Water and acetonitrile (HPLC grade) were obtained from J.T. Baker (Phillipsburg, NJ, USA). Pooled human liver S9 (HLS9) fraction, pooled male ICR mouse liver S9 (MLS9) fraction, pooled human liver microsome (HLM), and pooled male ICR mouse liver microsome (MLM) were purchased from XenoTech, LLC. (Kansas City, KS, USA). Pooled human plasma and pooled male ICR mouse plasma were purchased from Innovative Research, Inc. (Peary Court Novi, MI, USA). Potassium phosphate buffer (0.1 M, pH 7.4) was procured from Biosesang, Co., Ltd. (Seongnam, Gyeonggi-do, Republic of Korea). Phosphate-buffered saline (pH 7.2; PBS) was purchased from Corning Korea Co., Ltd. (Gangnam-gu, Seoul, Republic of Korea).

### 2.2. Pharmacokinetic Studies in Male ICR Mice

All animal procedures were reviewed and approved by the Institutional Animal Care and Use Committee (IACUC) of Korea University (Approval No: KUIACUC-2020-0031) and conducted in compliance with the IACUC guidelines. Male ICR mice (8 weeks old, 30–35 g body weight) were obtained from Koatech (Pyeongtaek, Gyeonggi-do, Republic of Korea) and acclimated for at least one week under controlled temperature (22 ± 2 °C), humidity (50 ± 10%), and a 12 h light/dark cycle, with ad libitum access to standard chow and water. In accordance with an approved protocol, mice were euthanized by CO_2_ asphyxiation at the end of the study.

For intravenous (IV) administration, KB-1517 was dissolved in a vehicle of dimethylacetamide/Tween 80/water (5/5/90, *v*/*v*/*v*) at 1 mg/mL. KB-1518 (as TFA salt) was dissolved in DMSO/saline (10/90, *v*/*v*) at 1 mg/mL. Mice (n = 4 per group per compound) received a single IV dose of 5 mg/kg via the tail vein (dose volume: 5 mL/kg). For oral (PO) administration, the same formulations were used, and mice (n = 4 per group per compound) received a single dose of 10 mg/kg via oral gavage (dose volume: 10 mL/kg). For intraperitoneal (IP) administration, a suspension of KB-1517 or KB-1518 (0.4 mg/mL in PBS) was prepared by diluting a DMSO stock solution (13 mg/mL). Mice (n = 4 per compound) received a single IP injection of 0.2 mg per animal (dose volume: 500 µL).

Serial blood samples (approximately 40 µL) were collected via the saphenous vein into Microvette^®^ 100LH heparinized capillary tubes at selected time points. Blood samples were immediately centrifuged (6000× *g*, 5 min, 4 °C) to obtain plasma. Plasma samples were stored in freezer until LC-MS/MS analysis.

Protein precipitation was performed on 15 µL aliquots of plasma samples by adding three volumes of acetonitrile containing diclofenac as an analytical internal standard. The mixture was vortexed thoroughly and centrifuged at 3000× *g* for 30 min at 4 °C. A 50 µL aliquot of the resulting supernatant was transferred to a 96-well plate and mixed with 50 µL of water. The prepared solutions were analyzed using an Agilent 6460 QQQ LC-MS/MS system (Agilent Technologies Korea Ltd., Seoul, Republic of Korea).

### 2.3. In Vitro Metabolic Stability in Liver Tissue Fractions

Metabolic stability was assessed using pooled HLS9 and MLS9 fractions. The S9 fractions (20 mg/mL) were diluted to 1 mg/mL with 0.1 M potassium phosphate buffer (pH 7.4). Stock solutions of KB-1517 and KB-1518 (10 mM in DMSO) were diluted in 50% aqueous acetonitrile to prepare working solutions (120 µM). A Tris mix buffer containing 1 M Tris-HCl (pH 7.4), 10 mM MgCl_2_, and 125 µg/mL alamethicin was prepared. A combined cofactor solution was prepared in 0.1 M potassium phosphate buffer (pH 7.4): 10 mM NADPH, 5 mM UDPGA, 1 mM PAPS, and 25 mM GSH.

The incubation mixture (final volume 200 µL) contained: 125 µL of deionized water, 40 µL of Tris mix buffer, 10 µL of 20 mg protein/mL S9 fraction (final concentration 1 mg protein/mL), and 5 µL of the test compound working solution (final concentration 3 µM). The final concentrations of DMSO and acetonitrile were 0.03% and 1%, respectively. The mixture was pre-incubated at 37 °C for 5 min in a shaking water bath. The reaction was initiated by adding 20 µL of the combined cofactor solution (final concentrations: 1 mM NADPH, 0.5 mM UDPGA, 0.1 mM PAPS, 2.5 mM GSH). Control incubations were performed without the cofactor mixture (replaced with buffer) and at 0 min (quenched immediately after adding cofactors). Incubations were carried out at 37 °C for 0, 15, 30, and 60 min (for stability) or 60 min (for metabolite ID). Reactions were terminated by adding 200 µL of ice-cold acetonitrile containing IS (diclofenac).

Terminated samples were sonicated (5 min), vortexed (1500 rpm, 5 min), and centrifuged (3000 rpm, 20 min, 4 °C). An aliquot (150 µL) of the supernatant was transferred to a 96-well plate for LC-MS/MS analysis as described in Section 2.6.2.

To determine the potential involvement of flavin-containing monooxygenase (FMO), a parallel assay was conducted with HLM and MLM [31]. To selectively inactivate FMO, microsomes (20 mg protein/mL) were pre-incubated at 45 °C for 5 min. Non-inactivated microsomes served as the control. Subsequently, metabolic incubations were performed with both control and heat-inactivated microsomes at 37 °C for 60 min. The incubation procedure was identical to that described above for the S9 fractions, with the exception that NADPH was used as the sole cofactor.

### 2.4. Metabolite Identification

Metabolite identification was performed using samples generated from the in vitro liver S9 incubations (final substrate concentration 20 µM, 60 min incubation) and plasma samples from the in vivo IP administration study. Samples were analyzed using an Agilent 6530 Q-TOF LC-MS/MS system (Agilent Technologies Korea Ltd., Seoul, Republic of Korea). Data processing and metabolite identification were performed using Agilent MassHunter Metabolite ID software (ver. B.04.00), searching for expected biotransformations (e.g., oxidation, demethylation, glucuronidation, sulfation, GSH conjugation) and comparing MS/MS fragmentation patterns of metabolites with the parent drug.

### 2.5. Binding Assays

The nonspecific binding of KB-1517 and KB-1518 in human and mouse plasma and liver S9 fractions was determined by equilibrium dialysis using a Rapid Equilibrium Device (RED) system (Thermo Fisher Scientific, Seoul, Republic of Korea).

Working solutions (200 μM for plasma, 60 μM for S9 fractions) were prepared in 40% aqueous acetonitrile. These were diluted 20-fold into either plasma or S9 incubation buffer (1 mg/mL protein, 200 mM Tris-HCl, 2 mM MgCl_2_, pH 7.4) to create the final dosing solutions.

For the dialysis, RED inserts (8 kDa MWCO) were used. Into each device, 200 μL of a dosing solution was added to the sample chamber, and 350 μL of PBS was added to the buffer chamber. The plate was sealed and incubated at 37 °C for 4 h on an orbital shaker (100 rpm) to allow the system to reach equilibrium.

Following incubation, 50 μL aliquots were taken from both chambers. To ensure analytical consistency, the samples were matrix-matched: the sample chamber aliquot was mixed with 50 μL of PBS, and the buffer chamber aliquot was mixed with 50 μL of the corresponding blank matrix (plasma or S9 incubation buffer). Proteins were then precipitated by adding 200 μL of ice-cold acetonitrile containing diclofenac (1 μg/mL) as IS. The samples were subsequently vortexed, sonicated for 5 min, and centrifuged at 4000× *g* for 20 min. Finally, a 100 μL aliquot of the resulting supernatant was diluted with 100 μL of HPLC-grade water in a 96-well plate for subsequent LC-MS/MS analysis.

### 2.6. Analytical Methods

#### 2.6.1. Quantitative Analysis

Quantitative analysis was performed using an Agilent 1290 Infinity Series HPLC system (Agilent Technologies Korea Ltd., Seoul, Republic of Korea) coupled to an Agilent 6460 QQQ mass spectrometer system (Agilent Technologies Korea Ltd., Seoul, Republic of Korea) with a dual AJS ESI ion source operating in positive ion mode. For chromatographic separation, an Agilent Eclipse Plus C18 column (2.1 × 100 mm, 3.5 µm) (Agilent Technologies Korea Ltd., Seoul, Republic of Korea) protected by a Phenomenex SecurityGuard C18 guard column was used. The mobile phase consisted of (A) 0.1% formic acid in water and (B) 0.1% formic acid in acetonitrile. A gradient elution was used: 5% B (0–2 min), 5–95% B (2–3 min), 95% B (3–7 min), followed by re-equilibration. The flow rate was 0.45 mL/min, the injection volume was 3 µL, and the column temperature was 40 °C.

The ion source parameters for the mass spectrometer were set as follows: gas temperature 325 °C, gas flow 12 L/min, nebulizer pressure 35 psi, sheath gas temperature 350 °C, and sheath gas flow 10 L/min. Multiple Reaction Monitoring (MRM) transitions were used for quantification: KB-1517: *m*/*z* 402 → 72 (CE 10 V); KB-1518: *m*/*z* 402 → 72 (CE 5 V); Diclofenac (IS): *m*/*z* 296 → 215 (CE 30 V); Propranolol: *m*/*z* 260 → 56 (CE 21 V); Ranitidine: *m*/*z* 315 → 102 (CE 33 V); Itraconazole: *m*/*z* 705 → 395 (CE 40 V); Antipyrine: *m*/*z* 189 → 56 (CE 26 V). Data acquisition and processing were performed using Agilent MassHunter Quantitative Analysis software (ver. B.05.00). Calibration curves were constructed using weighted linear regression.

#### 2.6.2. Qualitative Analysis

Qualitative analysis for metabolite identification was performed using the same LC system coupled to an Agilent 6530 Q-TOF mass spectrometer system (Agilent Technologies Korea Ltd., Seoul, Republic of Korea) with a dual AJS ESI source operating in positive ion mode. The chromatographic conditions were identical to those used for quantitative analysis.

The ion source parameters for the mass spectrometer were set as follows: gas temperature 325 °C, gas flow 12 L/min, nebulizer pressure 35 psi, sheath gas temperature 350 °C, and sheath gas flow 10 L/min. Data were acquired in MS scan mode (*m*/*z* 100–1000) and targeted MS/MS or Auto MS/MS mode. Data acquisition and processing were performed using Agilent MassHunter Qualitative Analysis (ver. B.05.00) and Metabolite ID software (ver. B.04.00).

### 2.7. Data Analysis

Noncompartmental analysis (NCA) was performed using PKSolver to determine pharmacokinetic parameters from the plasma concentration-time data. Parameters included: maximum plasma concentration (C_max_), time to reach C_max_ (t_max_), terminal elimination half-life (t_1/2_), area under the plasma concentration-time curve from time zero to the last measurable concentration (AUC_last_), area under the plasma concentration-time curve extrapolated to infinity (AUC_inf_), total body clearance (CL), and volume of distribution at steady state (V_ss_). Oral bioavailability (F) was calculated as: F(%) = (AUC_inf,PO_/AUC_inf,IV_)× (Dose_IV_/Dose_PO_)×100. Due to the anomalous profile of KB-1517, AUC_last_ was used for its F calculation, and some parameters (t_½_, CL, V_ss_ for IV; AUC_inf_ for PO) could not be reliably determined (ND). The metabolic rate constant (k) was determined by fitting the in vitro metabolic stability data to a one-phase decay equation via nonlinear regression using GraphPad Prism (Version 10, GraphPad Software, Boston, MA, USA). The in vitro t_1/2_ was calculated from the equation t_1/2_ = ln(2)/k. Subsequently, the unbound intrinsic clearance (CL_int,u_) was calculated as follows: CL_int,u_ = k/(f_u,S9_ × [S9]), where f_u,S9_ is the fraction unbound in the S9 incubation and [S9] is the S9 protein concentration.

## 3. Results

### 3.1. Pharmacokinetics of KB-1517 and KB-1518 in Mice

**Figure 2:**
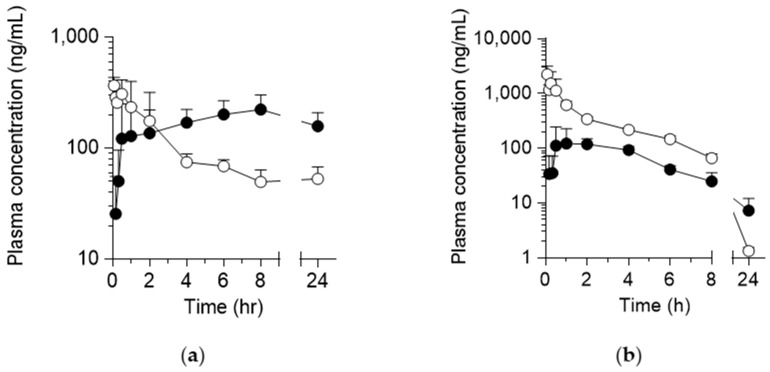
Plasma concentration-time curves of KB-1517 (**a**) and KB-1518 (**b**) following a single IV (5 mg/kg; ○) and PO (10 mg/kg; ●) dose in mice. Data are mean ± SD (n = 4).

The plasma concentration-time profiles of KB-1517 and KB-1518 following single IV (5 mg/kg) and PO (10 mg/kg) administration in male ICR mice are shown in Figure 2, and the derived pharmacokinetic parameters are summarized in Table 1.

Following IV administration, KB-1517 exhibited an unusual pharmacokinetic profile, characterized by an unexpectedly sustained plasma concentration observed between 8 and 24 h time points (Figure 2a). This prevented the accurate determination of the terminal elimination phase, rendering the calculation of terminal t_½_, CL, and V_ss_ unreliable (Table 1, ND). After PO administration, KB-1517 demonstrated slow absorption, with plasma concentrations gradually increasing and reaching C_max_ at a late t_max_ of 5.6 ± 3.5 h (Table 1). The apparent oral F, calculated using AUC_last_ due to the inability to determine AUC_inf_ reliably, was 119.3 ± 20.1%.

In contrast, KB-1518 displayed more conventional pharmacokinetic behavior (Figure 2b, Table 1). Following IV administration, KB-1518 showed a bi-phasic decline with a terminal t_½_ of 2.6 ± 0.3 h. The CL was relatively low at 1.5 ± 0.2 L/h/kg, and the V_ss_ well exceeded total body water at 4.6 ± 1.4 L/kg, indicating extensive distribution into tissues. After PO administration, KB-1518 was absorbed with a t_max_ of 1.6 ± 0.8 h and exhibited a longer apparent terminal t_½_ of 6.2 ± 1.9 h compared to the IV route, possibly due to flip-flop kinetics (absorption rate-limited elimination) or continued distribution. However, the oral F was low, calculated as 13.6 ± 3.4%, suggesting either poor absorption from the gastrointestinal tract or significant first-pass metabolism.

Both KB-1517 and KB-1518 exhibited high protein binding, with an unbound fraction (f_u_) of less than 2% in both human and mouse plasma (Table 2). KB-1518 also appears to have high tissue binding, as indicated by its high V_ss_ despite the high plasma protein binding in mice.

### 3.2. In Vitro Metabolic Stability of KB-1517 and KB-1518

The metabolic stability of KB-1517 and KB-1518 was evaluated by incubating the compounds (3 µM) with HLS9 and MLS9 fractions (1 mg/mL) in the presence of a comprehensive set of phase I and phase II cofactors (NADPH, UDPGA, PAPS, and GSH) for up to 60 min.

**Figure 3:**
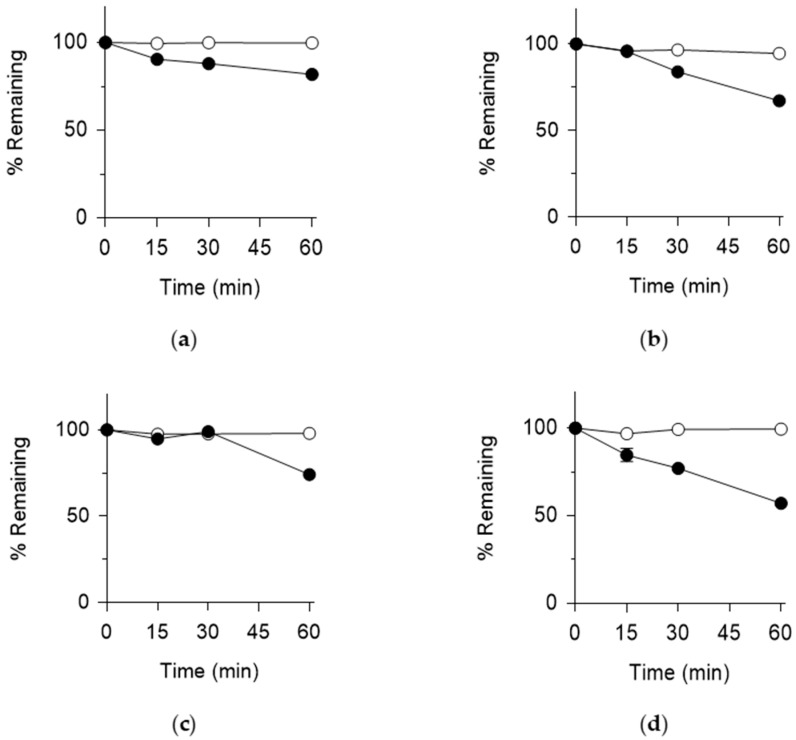
Metabolic stability of KB-1517 (**a**,**b**) and KB-1518 (**c**,**d**) in HLS9 (**a**,**c**) and MLS9 (**b**,**d**) fractions. % Remaining was calculated relative to the 0-min control in the absence (○) and presence (●) of cofactors (NADPH, UDPGA, PAPS and GSH). Data are mean ± SD (n = 3).

KB-1517 demonstrated high metabolic stability in both species. In HLS9, 81.9 ± 0.7% remained after 60 min, while 67.2 ± 1.0% remained in MLS9 (Figure 3), with an estimated t_½_ of 185.3 and 183.4 min in HLS9 and MLS9, respectively.

KB-1518 also exhibited considerable metabolic stability. In HLS9, 74.2 ± 2.5% remained after 60 min (Figure 3), corresponding to an estimated t_½_ of 112.6 min. In MLS9, KB-1518 showed slightly lower stability, with 57.2 ± 1.3% remaining after 60 min and an estimated t_½_ of 74.5 min (Figure 3).

Control incubations lacking cofactors showed minimal degradation for both compounds (Figure 3), confirming that the observed metabolism was cofactor-dependent. Overall, both KB-1517 and KB-1518 are relatively stable towards metabolism in liver S9 fractions, consistent with potentially longer in vivo half-lives, although KB-1518 appears slightly more susceptible to metabolism in mouse liver compared to human liver or KB-1517.

Both KB-1517 and KB-1518 showed high binding to human and mouse liver S9 fractions (f_u_ < 20%, Table 3). The incorporation of these binding data revealed a more pronounced difference in metabolic stability between the two compounds. The CL_int,u_ values for KB-1517 were 19.6 and 25.0 μL/min/mg protein in HLS9 and MLS9, respectively, which were significantly lower than those for KB-1518 (100.9 and 216.4 μL/min/mg protein in HLS9 and MLS9, respectively).

### 3.3. Metabolite Identification

To elucidate the metabolic pathways, metabolite identification studies were conducted using in vitro liver S9 fractions incubations (20 µM substrate) and in vivo plasma samples from mice administered an IP dose (200 µg/mouse). Metabolites were identified based on accurate mass measurements, isotopic patterns, and MS/MS fragmentation patterns compared to the parent compound using LC-Q-TOF MS/MS system (Agilent Technologies Ltd., Seoul, Republic of Korea).

#### 3.3.1. Identification of KB-1517 Metabolites In Vitro

**Figure 4:**
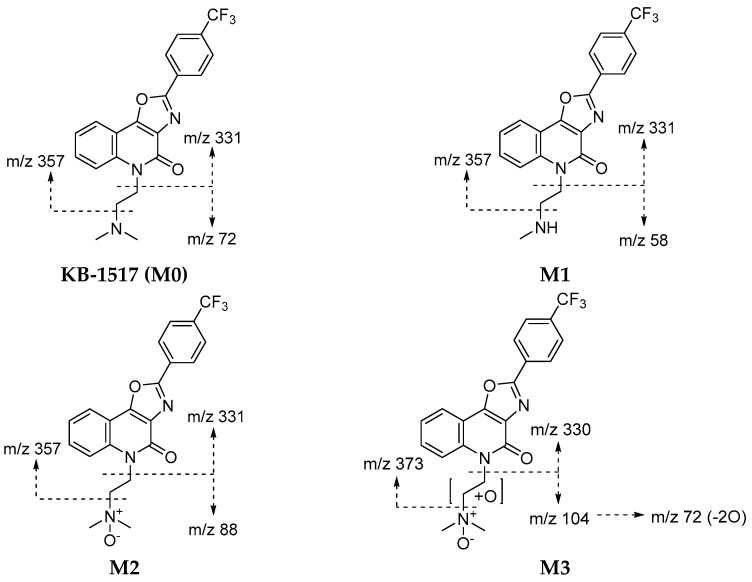
Proposed structures and mass fragmentation patterns of putative KB-1517 metabolites identified using MLS9 and HLS9 fractions. KB-1517 metabolites were identified as described in Table 4.

**Scheme 1:**
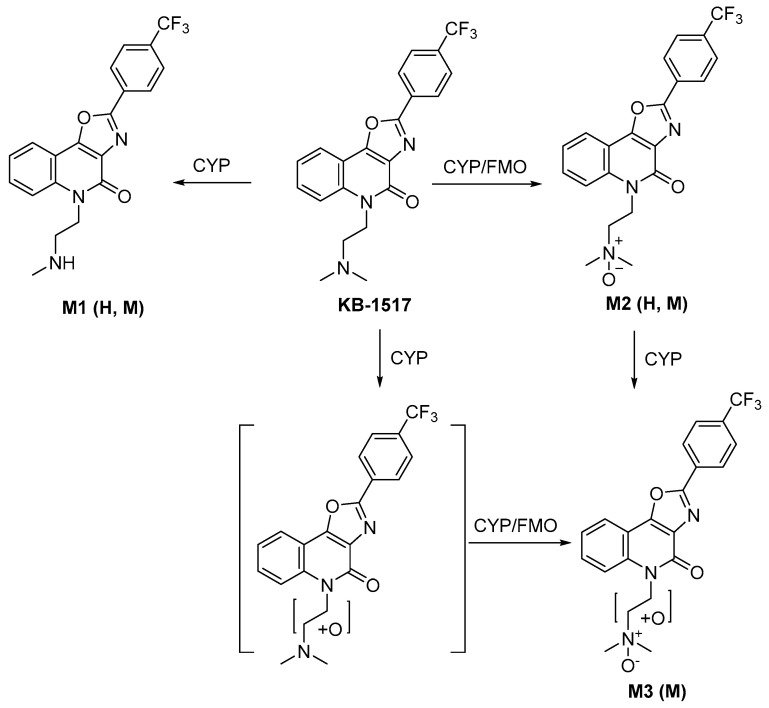
Proposed metabolic pathway of KB-1517 in liver S9 fractions. H: human. M: mouse.

Incubation of KB-1517 with HLS9 and MLS9 fractions in the presence of cofactors yielded three primary metabolites, designated M1, M2, and M3 (Table 4). Proposed structures and fragmentation patterns are shown in Figure 4, and the proposed metabolic pathway is depicted in Scheme 1.

Metabolite M1 ([M+H]^+^ *m*/*z* 388.1267) was identified as a mono-demethylated product, consistent with the loss of a methyl group (−14 Da) from the dimethylaminoethyl side chain. Its MS/MS spectrum showed characteristic fragment ions, including *m*/*z* 58 (loss of one methyl from the *m*/*z* 72 fragment of the parent). M1 was detected in both HLS9 and MLS9 incubations.

Metabolite M2 ([M+H]^+^ *m*/*z* 418.1373) corresponded to mono-oxidation (+16 Da) of KB-1517. Its MS/MS spectrum featured a prominent ion at *m*/*z* 88 (*m*/*z* 72 + O) and retained the core fragment ions (*m*/*z* 331, 357), suggesting oxidation occurred at the tertiary amine of the dimethylamino group. M2 was the most abundant metabolite detected in both HLS9 and MLS9.

Metabolite M3 ([M+H]^+^ *m*/*z* 434.1323) was identified as a di-oxygenated product (+32 Da). Its MS/MS spectrum included fragment ions at *m*/*z* 104 (*m*/*z* 72 + 2O) and *m*/*z* 330, suggesting potential sequential oxidation or hydroxylation at two different sites of the dimethylaminoethyl side chain; the fragment ion at *m*/*z* 373 (*m*/*z* 357 + O) further suggests that M3 was formed by N-oxidation plus hydroxylation of the ethylene moiety of the side chain. M3 was detected only in MLS9 incubations, indicating a species-specific metabolic route in mice.

These findings suggest that KB-1517 primarily undergoes phase I oxidative metabolism via N-oxidation and N-demethylation, with N-oxidation likely being the predominant pathway in vitro.

#### 3.3.2. Identification of KB-1518 Metabolites In Vitro

**Figure 5:**
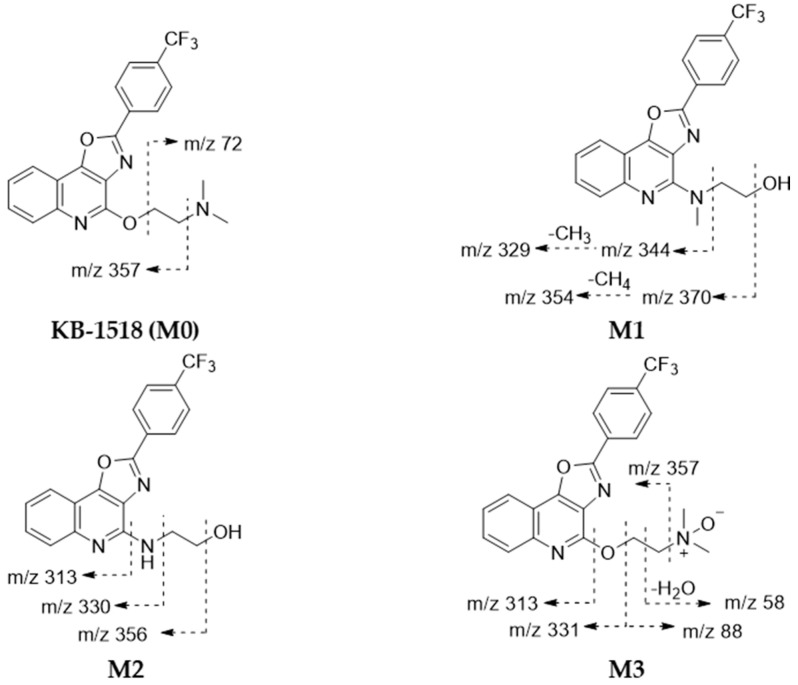
Proposed structures and mass fragmentation patterns of putative KB-1518 metabolites identified using MLS9 and HLS9 fractions. KB-1518 metabolites were identified as described in Table 5.

**Scheme 2:**
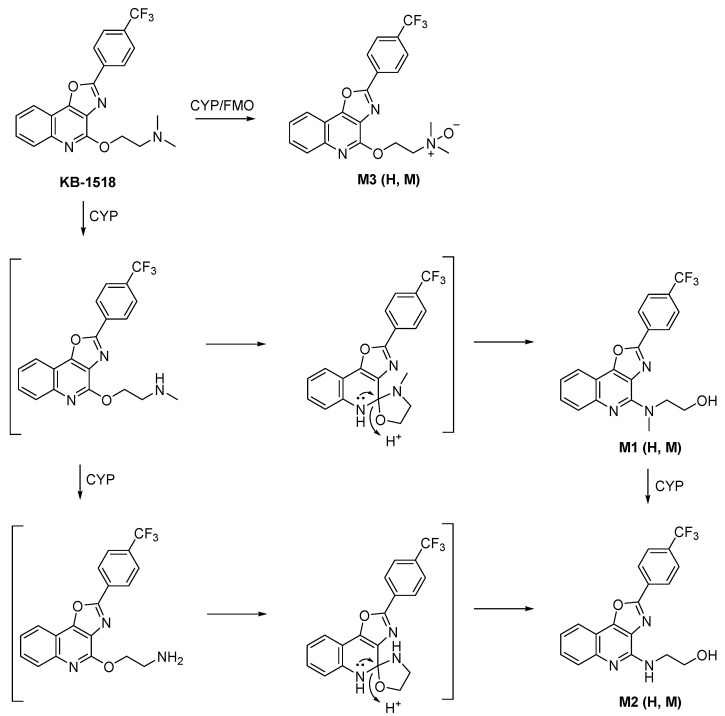
Proposed metabolic pathway of KB-1518 in liver S9 fractions. H: human. M: mouse.

Incubation of KB-1518 yielded three main metabolites (M1, M2, and M3) in both HLS9 and MLS9 fractions (Table 5). Proposed structures and pathways are shown in Figure 5 and Scheme 2.

Metabolite M1 ([M+H]^+^ *m*/*z* 388.1261) was identified as a metabolite formed by rearrangement of the N-mono-demethylated side chain (−14 Da) of KB-1518. Its MS/MS spectrum showed characteristic fragment ions, including *m*/*z* 370 (loss of H_2_O from the side chain of the parent) and *m*/*z* 344 (loss of hydroxyethyl from the side chain of the parent). Similarly, metabolite M2 ([M+H]^+^ *m*/*z* 374.1109) was identified as a metabolite formed by rearrangement of the N-di-demethylated side chain (−28 Da) of KB-1518. Its MS/MS spectrum showed characteristic fragment ions, including *m*/*z* 356 (loss of H_2_O from the side chain of the parent) and *m*/*z* 330 (loss of hydroxyethyl from the side chain of the parent). Metabolite M3 ([M+H]^+^ *m*/*z* 418.1373) corresponded to mono-oxidation (+16 Da) of KB-1518. Its MS/MS spectrum displayed characteristic fragment ions consistent with N-oxidation of the side chain, such as *m*/*z* 357 (loss of N,N-dimethylamine-N-oxide from the side chain) and *m*/*z* 88 (mono-oxidated side chain). The three metabolites of KB-1518 putatively identified were detected in both HLS9 and MLS9 incubations in this study. The relative abundance of these metabolites differed between species: M1 > M3 > M2 in HLS9 fraction, whereas M3 > M1 > M2 in MLS9 fraction.

These results suggest that KB-1518 also primarily undergoes phase I oxidative metabolism via N-oxidation and N-demethylation (+side chain rearrangement).

#### 3.3.3. Involvement of FMO in the Metabolism of KB-1517 and KB-1518

To investigate the involvement of (FMO in the metabolism of KB-1517 and KB-1518, their metabolic stability was evaluated in vitro using HLM and MLM. The experiments were conducted with and without prior heat inactivation (45 °C for 5 min), a method used to selectively inhibit FMO activity [31].

**Figure 6:**
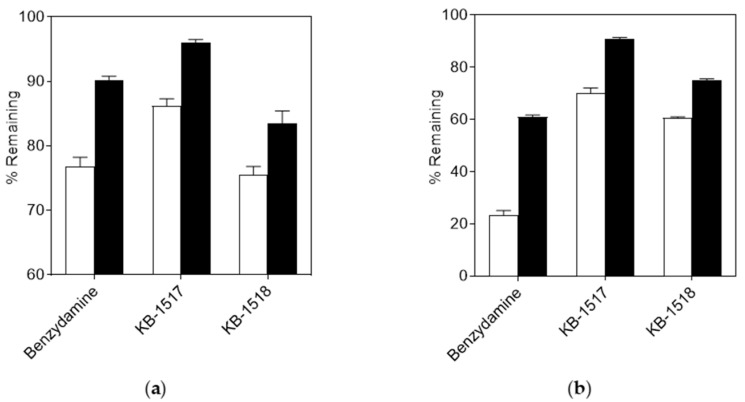
Effect of heat inactivation on the metabolic stability of KB-1517 and KB-1518 in HLM (**a**) and MLM (**b**). % Remaining was determined after a 60-min incubation and calculated relative to the 0-min control. Open and filled bars represent reactions conducted with active and heat-inactivated (45 °C for 5 min) S9 fractions, respectively. Benzydamine was included as a positive control. Data are mean ± SD (n = 3).

As expected, heat inactivation significantly inhibited the metabolism of benzydamine, a well-known FMO substrate, in both HLM and MLM (Figure 6), consistent with previous report [31]. A similar inhibitory effect was observed for both KB-1517 and KB-1518, suggesting that FMO contributes, at least in part, to their biotransformation (Figure 6). Furthermore, these results imply that the N-oxide metabolites of the test compounds identified in this study were likely formed, at least partially, through FMO-mediated pathways [32].

#### 3.3.4. Identification of Metabolites In Vivo

**Figure 7:**
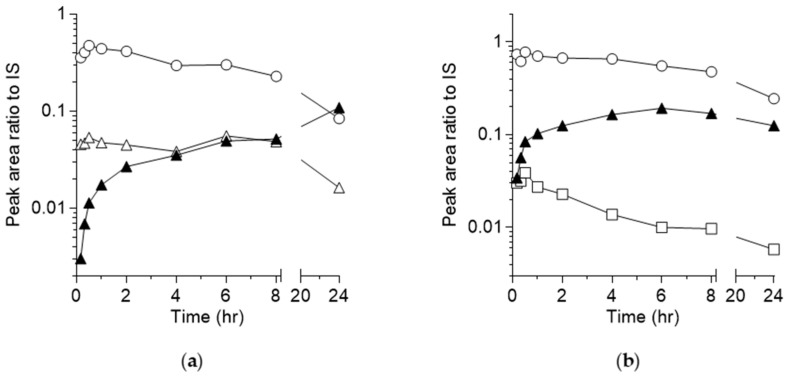
Time course of KB-1517 (**a**), KB-1518 (**b**) and detected metabolites (M0: ○; M1: ▲, M2: △; M3: □) in mouse plasma following a single IP injection in mice. Note: the peak area ratios do not indicate the relative levels of the analytes, as these ratios are dependent on the ionization efficiencies of the compounds in LC-MS analysis.

Analysis of pooled plasma samples from mice administered KB-1517 via IP injection revealed the presence of the parent drug and two major metabolites corresponding to M1 (mono-demethylation) and M2 (N-oxidation) identified in vitro. The time course of their relative abundance (peak area ratio to IS) is shown in Figure 7a.

The parent concentration decreased with time over the time course. The M1 concentration steadily increased over the 24-h period, suggesting continuous formation and potentially slower elimination compared to the parent, becoming the predominant circulating metabolite at later time points. M2 was formed rapidly, peaked early at t_max_ similar to that of the parent, and then declined, although its concentration remained relatively stable between 2 and 8 h before decreasing by 24 h. This suggests M2 is formed but possibly cleared more rapidly or further metabolized compared to M1 in vivo.

Similarly, analysis of pooled plasma from mice dosed IP with KB-1518 identified the parent drug and two major metabolites corresponding to M1 (mono-demethylation + rearrangement) and M3 (N-oxidation) observed in vitro. The time course is shown in Figure 7b. The parent concentration declined steadily over 24 h. The M1 concentration increased, peaking around 6 h, and then gradually decreased, suggesting continuous formation and potentially slower elimination compared to the parent, becoming the predominant circulating metabolite at later time points. M3 appeared rapidly after administration but its concentration decreased relatively quickly over time, indicating faster elimination or further metabolism compared to M1 in vivo.

## 4. Discussion

This study provides the first comprehensive characterization of the pharmacokinetic and metabolic profiles of KB-1517 and KB-1518, new oxazolo[4,5-*c*]quinoline-based small-molecule inhibitors targeting the IL-33/ST2 signaling pathway. Understanding the ADME properties is crucial for evaluating the therapeutic potential and guiding the development of new chemical entities.

The in vivo pharmacokinetic studies in mice revealed distinct profiles for the two analogs. KB-1517 displayed sustained plasma concentration at later time points post-IV dose, indicating significant enterohepatic recirculation or complex distribution phenomena involving slow release from tissue reservoirs. The delayed t_max_ after oral dosing is consistent with enterohepatic recirculation, prolonged distribution, or slow intestinal absorption. These characteristics suggest potentially prolonged exposure but also highlight complexities in its disposition that warrant further investigation and clarification.

KB-1518 exhibited more predictable pharmacokinetics following IV administration, with moderate tissue distribution (V_ss_ ~4.6 L/kg) and relatively low clearance (CL ~1.5 L/h/kg), consistent with its observed in vitro metabolic stability. However, its oral bioavailability was markedly low (~14%). This could be attributed to poor absorption from the gastrointestinal tract (potentially due to physicochemical properties like solubility or permeability) or extensive first-pass metabolism in the gut wall or liver [33]. The longer apparent half-life after oral dosing might suggest absorption-limited elimination (flip-flop kinetics) [34]. The low bioavailability presents a challenge for oral delivery and may necessitate formulation strategies (e.g., solubility enhancement) or exploration of alternative administration routes [35].

The in vitro metabolic stability studies using liver S9 fractions corroborated the in vivo findings to some extent, showing both compounds to be relatively stable, particularly KB-1517. The use of S9 fractions allows assessment of both phase I (e.g., CYP, FMO) and phase II (e.g., UGT, SULT, GST) metabolism. The primary routes identified involved phase I oxidative reactions.

Metabolite identification revealed N-oxidation and N-demethylation as key metabolic pathways for both compounds. For KB-1517, N-oxidation (M2) appeared dominant in vitro, while mono-demethylation (M1) led to the major circulating metabolite in vivo over time, suggesting potential differences in enzyme kinetics or clearance rates of the metabolites in vivo. The mouse-specific di-oxygenated metabolite (M3) highlights the importance of considering interspecies differences in metabolism. For KB-1518, N-demethylation followed by rearrangement (M1, M2) and N-oxidation (M3) were significant pathways both in vitro and in vivo. The difference in relative abundance between HLS9 and MLS9 fractions, and the distinct in vivo profiles of M1 and M3, further underscore the complexity of its metabolism and disposition. The identification of these metabolites provides crucial information for assessing potential pharmacologically active or toxic species and understanding drug–drug interaction potential mediated via metabolic enzymes (likely CYPs and/or FMOs). Further studies using specific enzyme inhibitors or recombinant enzymes would be needed to pinpoint the exact enzymes involved.

Overall, this study highlights KB-1517 and KB-1518 as promising leads with generally favorable metabolic stability but distinct pharmacokinetic challenges. KB-1517’s complex disposition requires further elucidation, while KB-1518’s low oral bioavailability needs to be addressed for optimal development.

## 5. Conclusions

This study characterized the pharmacokinetic and metabolic profiles of two new small-molecule IL-33 inhibitors, KB-1517 and KB-1518. Both compounds exhibited high metabolic stability in vitro, primarily undergoing phase I oxidative metabolism (N-oxidation, N-demethylation) in human and mouse liver S9 fractions. In vivo studies in mice revealed distinct pharmacokinetic characteristics: KB-1517 showed complex disposition with potentially prolonged exposure but anomalous kinetics requiring further investigation, while KB-1518 displayed moderate clearance and tissue distribution but had a low oral bioavailability. Metabolite profiling in vitro and in vivo confirmed the major metabolic pathways and identified key metabolites, revealing some species differences. These findings provide valuable ADME information, highlighting both the potential (stability, potential for sustained effect with KB-1517) and challenges (complex pharmacokinetics of KB-1517, low oral bioavailability of KB-1518) for the future development of these compounds as therapeutic agents targeting IL-33-mediated diseases. Further studies focusing on elucidating KB-1517’s kinetics, improving KB-1518’s oral absorption, assessing metabolite activity/toxicity, and investigating potential drug–drug interactions are warranted.

## Figures and Tables

**Table 1:** Pharmacokinetic parameters of KB-1517 and KB-1518 following a single IV and PO dose in mice.

Parameter	KB-1517		KB-1518	
	IV	PO	IV	PO
Dose (mg/kg)	5	10	5	10
t_1/2_ (h)	ND	ND	2.6 ± 0.3	6.2 ± 1.9
t_max_ (h)	NA	5.6 ± 3.5	NA	1.6 ± 0.8
C_max_ (ng/mL)	NA	289.0 ± 34.1	NA	156.8 ± 103.9
AUC_last_ (ng⋅h/mL)	1832.1 ± 348.1	4370.1 ± 738.3	3460.2 ± 474.5	868.4 ± 267.4
AUC_inf_ (ng⋅h/mL)	ND	ND	3464.6 ± 476.3	941.9 ± 234.0
CL (L/h/kg)	ND	NA	1.5 ± 0.2	NA
V_ss_ (L/kg)	ND	NA	4.6 ± 1.4	NA
F (%)	NA	119.3 ± 20.1	NA	13.6 ± 3.4

Pharmacokinetic parameters were calculated by noncompartmental analysis of the plasma con-centration-time curves presented in Figure 2. Values are mean ± SD (n = 4). NA: not applicable. ND: not determined.

**Table 2:** Plasma protein binding of KB-1517 and KB-1518.

Compound	f_u_ (%)	
	Human	Mouse
KB-1517	1.2 ± 0.03	1.8 ± 0.05
KB-1518	0.30 ± 0.01	0.25 ± 0.005
Propranolol	26.2 ± 2.6	24.7 ± 0.5
Ranitidine	85.0 ± 0.6	88.8 ± 5.4

f_u_ (%) was determined in triplicate in human and mouse plasma using equilibrium dialysis. Propranolol and ranitidine were included as high and low protein binder controls, respectively. Values are mean ± SD (n = 3).

**Table 3:** Binding of KB-1517 and KB-1518 in liver S9 fractions.

Compound	f_u_ (%)	
	Human	Mouse
KB-1517	19.1 ± 0.7	15.1 ± 0.8
KB-1518	6.1 ± 0.1	4.3 ± 0.5
Itraconazole	2.8 ± 0.6	1.9 ± 0.2
Antipyrine	94.6 ± 2.6	93.7 ± 1.1

f_u_ (%) was determined in triplicate in human and mouse liver S9 fractions using equilibrium dialysis. Itraconazole and antipyrine were included as high and low binder controls, respectively. Values are mean ± SD (n = 3).

**Table 4:** Mass spectral data of putative KB-1517 metabolites identified using MLS9 and HLS9 fractions.

No.	Biotransformation	Formula	[M+H]^+ a^ (*m*/*z*)	△m ^b^ (ppm)	t_r_^c^ (min)	Species	Product ions (*m*/*z*)
M0	Parent	C_21_H_18_F_3_N_3_O_2_	402.1425	0.2	3.96	NA	72, 114, 313, 331, **357**
M1	Mono-demethylation	C_20_H_16_F_3_N_3_O_2_	388.1267	0.0	3.92	H, M	58, 114, 313, 331, **357**
M2	N-oxidation	C_21_H_18_F_3_N_3_O_3_	418.1373	0.0	3.99	H, M	58, 88, 114, 313, 331, **357**
M3	Di-oxygenation	C_21_H_18_F_3_N_3_O_4_	434.1323	0.2	3.99	M	72, 104, 330, **373**

KB-1517 (20 µM) was incubated with MLS9 or HLS9 fractions (1 mg protein/mL) for 60 min at 37 °C in the presence of 1 mM NADPH, UDPGA and PAPS as cofactors. Putative metabolites were identified by a Q-TOF LC-MS/MS system run in a positive auto and target MS/MS mode. ^a^ Observed mass. ^b^ Deviation of the observed mass from theoretical mass. ^c^ Retention time on chromatograms. H: human. M: mouse. NA: not applicable. Bold: most abundant product ions.

**Table 5:** Mass spectral data of putative KB-1518 metabolites identified using MLS9 and HLS9 fractions.

No.	Biotransformation	Formula	[M+H]^+ a^ (*m*/*z*)	△m ^b^ (ppm)	t_r_^c^ (min)	Species	Productions (*m*/*z*)
M0	Parent	C_21_H_18_F_3_N_3_O_2_	402.1428	1.0	4.01	NA	**72,** 357
M1	Mono-demethylation + rearrangement	C_20_H_16_F_3_N_3_O_2_	388.1261	−1.5	4.03	H, M	315, **329**, 344, 354, 370
M2	Di-demethylation + rearrangement	C_19_H_14_F_3_N_3_O_2_	374.1109	−0.3	3.99	H, M	313, 330, **356**
M3	N-oxidation	C_21_H_18_F_3_N_3_O_3_	418.1373	1.7	4.03	H, M	58, **88**, 313, 331, 357

KB-1518 (20 µM) was incubated with MLS9 or HLS9 fractions (1 mg protein/mL) for 60 min at 37 °C in the presence of 1 mM NADPH, UDPGA and PAPS as cofactors. Putative metabolites were identified by a Q-TOF LC-MS/MS system run in a positive auto and target MS/MS mode. ^a^ Observed mass. ^b^ Deviation of the observed mass from theoretical mass. ^c^ Retention time on chromatograms. H: human. M: mouse. NA: not applicable. Bold: most abundant product ions.

## Data Availability

The data presented in this study are available on request from the corresponding author.
